# The Research Deficit and Expert Disagreement Regarding Music Selection for Psychedelic Assisted Therapy

**DOI:** 10.1021/acsptsci.5c00583

**Published:** 2025-09-16

**Authors:** Milo Moskovitz

**Affiliations:** 1801 Appalachian State University, Boone, North Carolina 28608, United States of America

**Keywords:** Psychedelic assisted therapy (PAT), Music, Lysergic acid diethylamide (LSD), Psilocybin

## Abstract

Prior research has determined that music plays a central role in psychedelic assisted therapy (PAT). While there is a general consensus of the importance of music during PAT, there are only three empirical studies published to date that directly investigate which type of music might best support PAT. Importantly, no review to date has critically analyzed these studies and identified the gaps. Careful examination reveals these studies have important limitations and the findings lack alignment with other publications and existing recommendations. Additionally, our understanding of guidelines seems to be not much different from when this research started in 1970. This paper summarizes the common impacts of music during PAT, reviews what we know about music selection and guidelines for PAT, and makes suggestions of priorities for future research.

## Graphical abstract

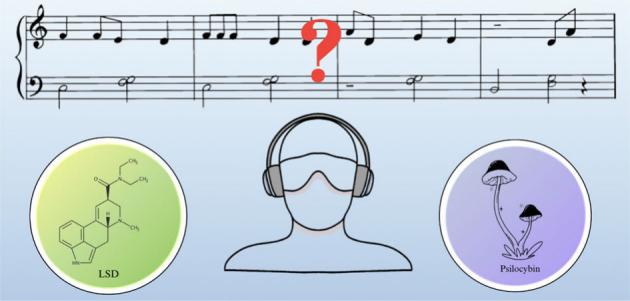

During the 1950s and 1960s, research into psychedelic therapy was thriving. However, this progress came to an abrupt halt in the early 1970s, largely due to the onset of the “war on drugs,” increased regulatory restrictions, and a significant decline in funding.^1^ Today, with growing evidence for the efficacy and safety of psychedelic-assisted therapy (PAT), a resurgence of psychedelic research is underway with music continuing to play a central role.^2^

Current research is showing great promise in treating a wide variety of psychological conditions including tobacco addiction,^3,4^ general addiction disorders,^5,6^ treatment resistant depression,^7–12^ obsessive-compulsive disorder,^13^ end of life depression and anxiety,^11,14^ and post-traumatic stress disorder.^15^ These disorders are often challenging — and in some cases, impossible — to treat effectively with conventional mental healthcare methods, making the effectiveness of just one or two PAT sessions all the more promising.

A distinctive quality of psychedelic therapy is that it may occasion a mystical experience, which is characterized as a profound feeling of oneness or unity.^16^ Recent research suggests that such experiences may have important therapeutic benefits.^11,14^ This is a unique pharmacological circumstance in which the drug effect is suggestible,^11,14,17^ meaning that the patient’s (mind)­set and setting can impact the therapeutic outcomes of the experience.^18^

One of the most common and impactful ways to create an optimal set and setting is the use of music.^19^ Nearly every published study regarding psychedelic-assisted therapy includes music with the aim of providing psychological support and potentially aiding in the occurrence of a mystical experience.^16^ Importantly, if a practitioner understands how music impacts the patient, they can direct them down the most therapeutically optimal path.

While there is a consensus regarding the importance of music during PAT, there is a severe lack of empirical research investigating which musical genres or qualities might be optimal. This scarcity of literature has changed little since the research began, as in 1970 Gaston and Eagle noted, “Thus far, in a number of hospitals, the selection for music for presentation in LSD sessions has been based on subjective opinion, generally of the psychiatrist in charge”.^20^ In this paper, I critically examine the empirical literature on music selection guidelines for PAT, identify the gaps, and provide suggestions of priorities for future research.

## Brief Overview of Music in PAT

To understand the experience of music during PAT, it is important to note the different phases of a psychedelic drug effect. Given the subjective and nonhomogeneous nature of psychedelic experiences, it is not surprising that the drug action phases have been delineated differently by many experts. While some models have subphases,^21^ and others do not,^22^ generally all models share three overall stages: an ascent, peak, and descent.

For the sake of clarity, the main impacts one might experience while listening to music during PAT have been categorized below into three key elements: journey, resonance, and reassurance, as delineated by Mendel Kaelen and colleagues.^2,23^

J*ourney* in a PAT session is the feeling of transcendence,^2^ being carried on a journey or given direction,^2,21,24^ and enhanced emotion, imagery, and meaning.^2,21,25,26^ Prior studies have demonstrated that listening to music during these PAT sessions can offer direction and continuity during an otherwise timeless experience.^2,21^ Additionally, Bonny and Phanke^2^ identified music as being continuously active throughout the sessions and, in their view, the main factor moving the therapeutic process forward. *Resonance* in the context of PAT refers to how well the musical selection aligns with the participants psychological state. It has been described as the degree to which the emotion of the individual is in harmony with the emotional nature of the music,^2^ the openness of the individual toward the music,^2,27^ and a general liking of the music.^2,28^ According to a meta-analysis of ten studies, music that was specifically considered “liked” was found to be integral for optimizing PAT experiences.^24^ Additionally, there is promising evidence to suggest that an individual’s resonance with the music played during PAT can increase the likelihood of positive clinical outcomes.^2^

To optimize resonance, it appears to be important to match the music with the drug effect timeline described above. Bonny and Phanke^21^ identified two key variables in musical resonance: the appropriateness of the music and the time at which it is played. This is important to note because music that optimally supports the ascent phase may not optimally support the peak phase.^16^

*Reassurance* is one of the most important effects of music during PAT as it offers psychological support to the participants. Crucially, Bonny and others found that when music aligns with the intensity of the drug’s effect (resonance), it can act as a support system.^20,21^ More specifically, music creates a “structured reality”^20^ that acts as an “anchor” to the material world for the patient in an otherwise overstimulating and otherworldly experience.^20,21^ Many metaphors have been used to describe the reassuring nature of music, such as “Something [···] to lean on” as stated by a study participant.^20^ Additionally, research has demonstrated that music promotes calmness^2^ and can be used as a tool to ease the patient smoothly into the drug effect.^21^ Lastly, music complements PAT by allowing the individual to surrender their normal controls, and explore the inward experience to a fuller degree.^21^

## Foundational Research for Music During PAT

Before discussing the empirical literature on music selection for PAT, it is important to address the nonempirical yet foundational work of two researchers. William Richards and Helen Bonny made important recommendations based on their expert judgment of large numbers of clinical experiences.

Wiliam Richards, one of the pioneers of psychedelic research and cofounder of the Johns Hopkins Center for Psychedelic and Consciousness Research, is a central figure in the field of music and PAT. Although Richards did not publish empirical research on musical choices, he stated that he has been selecting music and designing playlists for PAT since 1963.^29^ In his book, *Sacred Knowledge*: *Psychedelics and Religious Experiences*,^30^ Richards described the methodology for the included playlist creation as “carefully developed through trial and error and has been found to work well with many different people over time” (pp. 189–190). This playlist (developed with his son Brian Richards) has been used for contemporary research (e.g.,^31^) as a standard metric.

Helen Bonny created the music therapy method of Guided Imagery and Music (GIM), which while not made for the use of psychedelics, is often used as a template for musical decisions in PAT. Researchers have turned to GIM recommendations due to the lack of definitive guidelines for music during PAT as the two share important qualities like nonordinary states of consciousness and introspection.^32^ Bonny’s largest contribution to the field of music for PAT was her evaluation of over 600 PAT sessions at the Maryland Psychiatric Research Center.^21^ Bonny and her colleague, Walter Phanke, reviewed these sessions and determined important conclusions regarding the role of music during PAT. Because music selection itself was not a factor in the study, the musical recommendations are based on trial and error and observations.

## Empirical Literature for Music Selection

In spite of the clear importance of music during PAT sessions, there is little research examining what kinds of music might be optimal. To date, only three empirical published studies have directly investigated different music types to support PAT.

The first such study was conducted by Gaston and Eagle^20^ toward the end of the first wave of psychedelic research in 1970. Guided by early suggestions that music may be important during PAT, this experiment aimed to collect quantitative data regarding the function of music in lysergic acid diethylamide (LSD) therapy for patients with alcoholism. While their primary goal was to investigate the role of music during PAT, they attempted to gain insights into music preferences and preference changes.

Gaston and Eagle conducted the experiment during the high-dose psychedelic session at an ongoing LSD treatment program. A total of 59 patients were used as subjects. The study included five experimental conditions:

1. no music
2. miscellaneous music (randomly chosen music played without headphones)
3. familiar music without headphones
4. familiar music with headphones
5. unfamiliar music without headphones

Before and after the PAT sessions, each patient was asked to fill out the LSD Music Preference Questionnaire in which nine categories of music were listed: hymns (religious or sacred), rock ‘n’ roll, country western, jazz, love ballad, folk, march, light classical, and heavy classical. In this questionnaire, subjects ranked their preferences of the nine musical options.

By surveying the participants on their experience, the study draws valid conclusions about the benefits of music during PAT that align with findings from other literature on the subject (e.g., music as a tether to reality). While not the sole aim of the study, the researchers offer useful insights into the effect of music qualities. Researchers noticed participants preferred higher pitched music and found that generally, when the pitch was higher, participants saw brighter colors, smaller geometric shapes, and their memories of past experiences were more “real.” Additionally, low pitches were on average noticed more than high pitches. While important, these findings offer limited information about musical selection.

Gaston and Eagle attempted to measure musical preference and preference changes, but significant methodological issues and a lack of information undermine the study’s conclusions. One major problem arises with their definition of “familiarity” – pieces “selected according to each patient’s familiarity and liking for certain types of music.” A type or genre of music is a broad categorization that has many different kinds of music within it. One can be familiar with songs in a genre but completely unfamiliar with other songs in the same genre (e.g., rock music - the Beatles vs AC/DC). For the calibration of ″familiar music,” participants were asked to do three tasks: (1) indicate whether they were familiar or unfamiliar with each of the nine categories of music, (2) rank them based on preference, and (3) name a single example of a song from the familiar categories. Each of these tasks is problematic. First, familiarity cannot be a binary as it exists to varying degrees. Second, ranking music genres based on preference is not an accurate measure of familiarity, as ″familiarity″ and “preference” are not synonymous. Third, one example of a song is not enough for the researchers to necessarily select familiar music. These factors make it unlikely that all of the participants were indeed familiar with the specific songs played. With familiarity being a key variable measured in this study and the crux of many conclusions drawn, these serious flaws in methodology make the results unreliable.

Other problems arise with their conclusions, most notably that preference changes occurred more frequently among subjects exposed to familiar music through headphones. The conclusion the researchers drew from this discovery was, “that music familiar to the subjects and presented through stereophonic headphones was effective in producing changes in preferences for music.” However, they were not measuring the effectiveness in changing preferences, so this conclusion is illogical. Instead, this finding might suggest that solely relying on musical familiarity or preference does not optimally support PAT. This conclusion was not drawn by the researchers, and further testing would be needed to confirm this.

In addition to these methodological issues, other questionable aspects of this study are present. The authors made claims about musical preference: (a) Love ballads were more preferred by subjects in the *no music* group and (b) Love ballads were more preferred by subjects in the *miscellaneous music* group. However, this might be because those who preferred no music or miscellaneous music simply enjoyed the ease, sentimental, and melodic overtones that love ballads offer, regardless of psychedelic experience. It might be the case that those who selected no music, or random music simply prefer the least intrusive option being love ballads. Additionally, only one conclusion about music preference *changes* was statistically significant. This was that preference changes were more frequent for participants who heard familiar music through headphones – of which hymns and love ballads were more preferred and jazz less preferred. However, it is unclear whether these changes were related to the psychedelic experience itself. Because the LSD Music Preference Questionnaire did not include questions about prior headphone use, it is possible that this was the first instance in which participants heard hymns or love ballads through headphones. Given that this study was conducted in 1970, a time when personal access to diverse music was limited, prior exposure to such listening conditions cannot be assumed.

Finally, a number of important methodological details are omitted. Such details include how the participants were selected for each treatment condition, how the music was selected (i.e., whether that was from their limited collection or otherwise), and actual songs selected (i.e., titles, albums, or artists). Collectively, these omissions further detract from the reliability of the conclusions. Most importantly, the authors never explicitly state whether the comparisons of music preference is based on the participant’s speculation or actual preference. Given that they provide no information on cross-analyzing actual music preferences, one can only reasonably infer that these rankings are participants’ speculations. If this is indeed the case, such rankings may not accurately reflect actual preferences during PAT.

To summarize, in regard to findings about musical preference, this paper should be treated skeptically and cautiously used as data for creating PAT music guidelines.

The next study to investigate optimal musical qualities was done by Barrett and colleagues^16^ 47 years later, highlighting the almost half-century pause of psychedelic research. The aim of this study was to identify consistent qualities in music believed to support ascent (what Barrett et al. call “pre-peak”) and peak psychedelic drug effects during PAT. They did this by quantitatively and qualitatively analyzing musical recommendations from experienced providers.

An anonymous online survey was sent to practitioners of psilocybin-assisted therapy with experience in either a research or therapeutic setting. The survey was distributed via word of mouth to collect two to three song recommendations in each of two categories: (1) music they feel optimally supports the *ascent* drug phase and (2) music they feel optimally supports the *peak* drug phase. After refining the selections from the ten respondents to meet the appropriate criteria, 22 recommendations were collected for ascent music and 22 for peak music.

The qualitative analysis was conducted by three music theory experts who reviewed and analyzed present features (e.g., complexity, pulse, and vocal language) for both categories of songs. After conducting individual reviews, the three experts then compared and refined the results, creating a final table of musical and acoustic features. The quantitative analysis included objective features of music such as brightness, fullness, and tempo. Music information retrieval (MIR) methods were used to identify these features and assign factor scores for each song. These scores were then compared for each feature between the ascent and peak categories.

When combining the quantitative and qualitative results, Barrett and colleagues found consistent features in the music that were supportive for the peak drug phase: “regular, predictable, formulaic phrase structure and orchestration[;] a feeling of continuous movement and forward motion that slowly builds over time[;] and lower perceptual brightness when compared to pre peak music.” Brightness (“the metaphorical sharpness and colorfulness of the sound”) was the only dimension that differed quantitatively.

In regards to musical features supporting the *ascent* drug phase, a lack of consistency was found. The authors suggest that either there is no optimal musical quality or it has yet to be discovered.

Barrett et al. acknowledge that the small sample size and anonymous format slightly weakens the recommendations’ generalizability and reliability. Additionally, they also acknowledged there may have been overlap in practitioner training and the songs analyzed are merely recommendations from experienced practitioners and not tested to be effective. Lastly, the study was limited to psilocybin in order to minimize confounding variables which narrows the generalizability of other psychedelic options.

While this is a promising first step toward understanding optimal music selections, these findings alone are not sufficient grounds for determining PAT guidelines.

To date, the only fully randomized study of music genre to support PAT was conducted by Strickland and colleagues[Bibr ref31] (2020), three years after Barrett et al.[Bibr ref16] (2017). The aim of this study was to determine if the status quo of Western classical music for PAT holds any unique benefit, specifically for tobacco smoking cessation.

With a total of 10 participants, two musical playlists were tested: “classical” (Western classical music such as Brahms, Bach, Mozart, and Beethoven) and “overtone” (overtone based playlist including Tibetan singing bowls, gongs, didgeridoo, chimes, bells, sitar, and human voice overtone singing). In contrast to the classical playlist, the overtone playlist often lacked traditional music qualities like melody and rhythm. Two doses of psilocybin were tested: a lower dose of 20 mg/70 kg and a higher dosage of 30 mg/70 kg.

Each participant had two sessions of PAT, including one of each genre of music and one of each dosage of psilocybin. The combinations of these two variables were randomly assigned. For each session, once drug effects had resolved, participants were asked to complete evaluations in order to gauge mystical experiences (e.g., “feelings of unity and transcendence of space/time”) as well as experiences they might have been challenged by (e.g., “panic and feelings of losing sanity”).

The results for the Mystical Experience Questionnaire indicated a trend of higher overall scores in the overtone sessions compared to the classical sessions. However, these findings were not statistically significant. For the Challenging Experience Questionnaire, the authors found no clear differences between the overtone and the classical sessions.

Once the two sessions (and the respective questionnaires) were complete, an opportunity for a third session was presented in which the participants were allowed to select either of the musical options. Six out of the ten participants selected the overtone playlist, suggesting that there was no distinct preference for either of the genres.

Interestingly, of 4 participants who chose the classical option for the third session, only 2 remained abstinent from smoking by the end of the 6 and 12 month follow ups. To contrast, 5 out of the 6 participants who chose overtone music remained abstinent. Because the sample sizes are so low, we cannot draw any firm conclusions about musical genre and smoking cessation. With that being said, these results clearly do not support the status quo of Western classical music. Combining the lack of significant differences and the trends favoring the overtone playlist, the authors question the norm of classical music as it seems to have no unique benefit for PAT.

The authors acknowledge limitations of their study including the small sample size, lack of data on personal musical experience, and the “minor” overlap of roughly 25% of musical selections between the two playlists. The authors suggest the overlap would have minimal impact on the data as they occur toward the beginning and end of the sessions. Nevertheless, the authors are correct that this “perhaps limit[s] the ability to fully differentiate between the two genres”.

An additional methodological concern not addressed by the authors is the lack of clear definition and distinction between the two playlists. Some of the specific musical selections do not seem to fit either of the two categories. For example, *Here Comes the Sun* and *What a Wonderful World*, among others, would not be considered western classical music nor overtone-based music.

With that being said, the study is successful at examining the status quo of Western classical music against “something else.” While this single experiment is not enough to conclude that Western classical music holds no unique benefit, these data should not be ignored as they are a promising starting point for determining optimal music selection. Additionally, given that there is no theoretical reason to believe that Western classical music is uniquely beneficial for supporting PAT, this study begins to examine if such a genre exists.

The authors make it clear that this study is a first step and more research is needed to fully understand the importance of musical genres for PAT.

## Differing Opinions

Given the minimal published literature on musical selection for PAT, the lack of research creates uncertainty among the lead researchers in this field. There seems to be significantly differing opinions among the experts on three important guidelines: optimal musical qualities, individualization of music during PAT, and musical continuity. Table 1 summarizes the contrasting views below.

**Table 1:** Contrasting Views on Important PAT Music Guidelines

**Suggested practices**	**Helen Bonny**	**Mendel Kaelen**	**William Richards**	**Barrett et al., 2017**
Optimal music qualities	“Nonrepetitious in style and with an emphasis on variety in presentation”^21^	“Repetitive form”	n/a	“rhythmic, directional, and predictable music choice might be favorable”^16^ (referring to peak music)
		“Dynamic form”		
		“Sense of change sense of direction”^23^		
		“During the peak is where these reconstructive pieces of music are being played”^23^		
Individualization	n/a	It is “highly critical to work with music in a personalized fashion”^23^	“Generally, it is unwise in my judgment for therapists or experiencers to ‘play disc jockey’ as a session is unfolding”^29^	n/a
Continuity	“The music should be continuous with minimal interruption”^21^	“Having blocks of music that are 20–40 minutes long, so every block of music can end in a period of silence.”^23^	“keep periods of silence brief”^29^	n/a
	“It is very important to avoid unnecessary lapses into silence”^21^			

Regarding musical qualities, Helen Bonny suggests the music (not specific to peak period) should be “Nonrepetitious in style and with an emphasis on variety in presentation”.^21^ Other scholars recommended the musical qualities align with the drug effect phases. As discussed above, Barrett and colleagues^16^ found certain musical qualities present in peak period recommendations from experienced PAT practitioners. These qualities were “regular, predictable, formulaic phrase structure and orchestration, a feeling of continuous movement and forward motion that slowly builds over time”.^16^ Mendel Kaelen, a lead researcher in this area, also suggests that peak period music differs from the ascent phase, yet his recommendations contrast with Barrett’s findings. In a lecture on music during PAT, Kaelen suggests that peak period music be highly dynamic, a building of tension, and release.^23^

Regarding whether PAT playlists should be personalized, Richards believes that a more standardized approach to musical selection is optimal, stating “Generally, it is unwise in my judgment for therapists or experiencers to ‘play disc jockey’ as a session is unfolding.”.^29^ This contrasts Kaelen’s individualized approach as he believes it is “highly critical to work with music in a personalized fashion”.^23^ Kaelen recommends that providers “check in with your patient and get a sense of what’s going on – and then based on that knowledge, you decide [what the individual needs]”.^23^ To date, there is no published empirical research investigating standardized versus individualized music for PAT, making the optimal choice unclear.

As to musical continuity, Bonny and Phanke make their opinion clear stating “It is very important to avoid unnecessary lapses into silence” and “The music should be continuous with minimal interruption”.^21^ Richards agrees, recommending that, especially during the peak phase, one should “keep periods of silence brief”.^29^ In contrast, Kaelen recommends “Having blocks of music that are 20–40 min long, so every block of music can end in a period of silence.^23^ To date, only one study with two participants has investigated the use of silence during PAT, limiting the ability to develop guidelines in this regard.^33^

## The Copenhagen Music Program

In 2022, Messell and colleagues attempted to consolidate the existing recommendations to create an example playlist in hopes of inspiring future playlist creation.^36^ Because of the paucity of data on this subject, the authors turned to the field of music therapy to obtain guidance. The playlist structure was based largely on prior work done by Bonny, along with Preller and Vollenweide^26^ who investigated the different phases of the psychedelic drug effect.

Once the authors created a playlist structure to match the effect timeline of a typical dose of psilocybin, they began searching for pieces of music to fit their template. They chose music from existing playlists created for psychedelic research, ceremonial purposes (within the underground community), and GIM (Guided Imagery and Music, an introspective music therapy created by Bonny).

Examples of playlists created for psychedelic research include Richards’s Johns Hopkins playlist, Kaelen’s Imperial College London playlist, and Strickland’s overtone-based playlist as discussed above. Examples of playlists created for ceremonial purposes include Rosalind Watts’s *Psilodep Session 2* ^37^ and Michael Rasa’s *This Journey We Take*.^38^ The musical selections derived from the field of GIM were chosen from practical books by Denise Grocke^34^ and Bruscia and McShane.^35^

The authors considered musical selections that might fit the appropriate phases, and when deemed suitable, were critically analyzed for specific qualities (e.g., “nerve, sensitivity, soulfulness and a general authenticity”). Additionally, in accordance with GIM guidelines, popular songs and those with familiar language were excluded.

Once the musical choices for each phase (with subphases) were finalized, the authors sequenced the songs to create a meaningful order. In line with GIM principles, Messell and colleagues emphasized the varying purposes each song could serve (e.g., “the music could lead up to, prepare for, extend, give relief”). To delineate a specific order, a table was created to label unique musical features and music psychological qualities in relation to the adjacent songs. This processes created desired transitions between pieces, whether smooth or contrasting as appropriate. After the first playlist draft was complete, all four authors reviewed and refined the selections and sequencing.

The authors used Taxonomy of Therapeutic Music (TTM), a music intensity rating tool within GIM, to quantitatively determine if their playlist matched the drug intensity of a medium/high dose of psilocybin. The TTM consists of nine music intensity profiles ranging from *supportive* to *mystery and transformation*. The authors note that, while the playlist roughly aligns with the drug effect timeline, some outliers exist, possibly because TTM was created in the field of GIM in which no psychedelic drug is present. This distinction is important because, in GIM, music is the primary driver of the process, whereas in PAT, music both supports and anchors the drug effect. Therefore, the authors suggest these scores should be taken cautiously and that TTM could be altered to work more closely in line with PAT in the future.

As acknowledged by the authors, the Copenhagen Music Program is a propositional playlist which requires testing and validation with the goal of inspiring future examination and playlist creation.

To date, the only published test of the *Copenhagen Music Program* was a case study done by Ratkovic et al.^39^ According to the authors, the original aim of the study was not to evaluate the music program, but rather a “personal and professional exploration of the effect of a high-dose psilocybin session.” The study consisted of a single 3.5 g dose of psilocybin with the participant being an indigenous, experienced user of psychedelics, and a member of the author’s research team.

The reviewer had an overwhelmingly negative experience with the Copenhagen playlist, primarily criticizing the selections of European composers associated with colonization, which negatively affected her experience as an indigenous woman. The authors expressed that they felt this was due to white imperialism rooted in the experience.

However, as acknowledged by Ratkovic et al.,^39^ the creators of the Copenhagen music program make it clear that it is not intended to be a one size fits all playlist, but rather inspiration and a guideline for others to create their own. The authors of the Copenhagen Music Program article explicitly state, “The aim of the article is to inspire others in their endeavours to create music programs for psychedelic interventions”^36^ and “If a therapist chooses to apply music from cultures foreign to them, it is advised that the therapist familiarize themselves with the function and cultural meaning of the music pieces, not to inflict unwanted associations in the listener”^36^ and “When working with ethnic minorities or racial trauma, some authors have suggested that the music choice can amplify intercultural power dynamics in the therapeutic relationship.”^36^

Ratkovic and colleagues seemed to be more focused on critiquing the colonialist foundation of current science, instead of conducting careful scientific inquiry. It is important to note that this is the only "empirical" test of a (PAT) playlist, proving how scarce the scientific knowledge is on this topic.

Despite the flaws of this study, Ratkovic and colleagues bring up valid concerns about how ethnic minorities or those who have experienced racial trauma might have negative experiences to Western religious music choices. Personal backgrounds and cultural histories have not yet been explored in the literature.

## Conclusions

The purpose of this review was to critically examine the existing literature on music selection and guidelines for PAT. Based on the research discussed above, there are limited conclusions that can be drawn. Only three studies^16,20,31^ empirically investigate how different music types might support PAT. For this important, complicated, and vast area of pharmacology, these three studies investigate largely different aspects, highlighting how little is known. Additionally, the limitations of these studies, individually and collectively, make it difficult to draw many firm conclusions. Gaston and Eagle’s^20^ study was problematic to the point of unusable data in this regard; Strickland et al.^31^ was the only fully randomized study; both Strickland et al.^31^ and Barrett et al.^16^ are only applicable to psilocybin and had small sample sizes of 10.

The current playlists being used in research settings – notably those of Bonny, Richards, and Kaelen – have yet to be thoroughly and clinically tested, with Barrett et al.^16^ providing the only legitimate and empirical evaluation to date. Consequently, there is a clear lack of consensus, with even leading researchers expressing contradicting opinions on fundamental guidelines.

While the benefits of music during PAT are known, there still remains a significant gap in empirical guidance regarding *how* the music should be selected. Psychedelic therapy has the potential to be a frontier of mental health treatment and bring relief to countless individuals. However, without empirically based music selection, we risk neglecting one of its most important therapeutic elements. Given the current state of research, it seems unreasonable for anyone at this point to claim guidelines for musical selection for PAT are firmly based on scientific evidence.

The research to date neglects a potentially significant aspect of music and PAT: patient individuality. Given the variable and subjective nature of both listening to music *and* psychedelics, it is reasonable to assume that a patient’s age, cultural background, musical history, etc., might have a significant impact on their PAT music experience. The lack of understanding and need for research on this topic is highlighted by the opposing views of the experts Richards and Kaelen.

In addition, there is still a lack of empirical research investigating optimal music qualities and genres of music during PAT which has the potential to shape the sessions entirely. Future studies, building on the work of Barrett et al.^16^ and Strickland et al.,^31^ should focus on music qualities and genre to help researchers and practitioners understand optimal music selections for PAT.
